# Real-life experience with sorafenib for advanced and refractory desmoid-type fibromatosis

**DOI:** 10.2340/1651-226X.2024.40583

**Published:** 2024-08-04

**Authors:** Delphine Schampers, Alexander Decruyenaere, Celine Jacobs, Lore Lapeire

**Affiliations:** Department of Medical Oncology, Ghent University Hospital, Ghent, Belgium

**Keywords:** Desmoid, aggressive fribromatosis, sorafenib, targeted therapy, dose reduction

## Abstract

**Background:**

In recent years, there has been a change in the therapeutic landscape of desmoid-type fibromatosis (DF). Watchful waiting is now preferred over initial local treatments such as surgery and radiotherapy. Systemic treatment is considered for progressive or symptomatic disease. The aim of this study is to review real-life data on the use of sorafenib in DF.

**Methods:**

We established a retrospective dataset of patients treated with sorafenib in our centre, Ghent University Hospital, for progressive DF. Patient demographics, disease characteristics, response to therapy using Response Evaluation Criteria in Solid Tumours 1.1 criteria and toxicity according to CTCAE v5.0 were assessed.

**Results:**

Eleven patients with DF were treated with sorafenib between 2020 and 2024. Median treatment duration was 20.4 months (95% confidence interval [CI], 10.0-NR). 36.4% achieved partial response, 54.5% stable disease and 9.1% progressive disease. For three patients, the treatment is ongoing. The median time to objective response rate is 15.0 months (95% CI, 8.8-NR). The majority (81.8%) experienced grade 2 toxicity, and one third of patients grade 3 toxicity (36.4%). The most common all-grade adverse event was skin toxicity (hand-foot syndrome, pruritus and rash) (90.9%). Nine patients (81.8%) needed dose reduction with a median time to first reduction of 1.1 months (95% CI, 0.5-NR). One patient stopped treatment due to toxicity.

**Interpretation:**

Real-life data on the use of sorafenib in the treatment of DF is consistent with published data in clinical trial setting. Sorafenib is an effective treatment option for progressive DF although associated with significant toxicity and the need for rapid dose reduction.

## Introduction

Desmoid-type fibromatosis (DF) is a rare subtype of soft tissue tumour with an incidence of five to six cases per 1 million of the population per year [1, 2]. Approximately 85% of cases are sporadic and the remaining 15% occur in the context of familial adenomatous polyposis [2–5]. Although DF lacks the potential to metastasise, it can cause significant morbidity due to its destructive growth pattern [2, 6]. The approach to treating DF has changed in recent years as a result of the growing knowledge of its pathophysiology and variable clinical presentation as it has been observed that these tumours can spontaneously regress. Therefore, depending on the anatomical location, symptoms and extent of disease, either watchful waiting or active treatment may be chosen. Active treatment should only be considered in case of persistent progression or symptomatic disease in order to avoid overtreating patients whose DF may spontaneously regress [2, 4–6]. Available active treatment options include local treatment such as surgery, radiotherapy, ablation techniques and systemic treatment such as antihormonal therapy, non-steroidal anti-inflammatory drugs, cytotoxic chemotherapy, tyrosine kinase inhibitors (TKIs) and recently gamma-secretase inhibitors [6, 7]. Due to the rarity of the disease, most of these treatment options have been investigated in small, often retrospective trials. Large prospective, randomised trials are lacking [2, 4–8]. A phase 3, placebo-controlled, randomised trial was conducted to investigate the use of sorafenib an oral multitargeted receptor TKI. This trial included 87 patients who were randomised to receive treatment with sorafenib 400 mg daily or placebo [9]. The results showed that the risk of progression was seven times lower with sorafenib compared to placebo (HR 0.14). Progression-free survival (PFS), the primary endpoint of the study, was 89% at 1 year (95% confidence interval [CI], 80–99) in the sorafenib group and 46% (95% CI, 32–67) in the placebo group [9]. The objective response rate (ORR) for sorafenib was 33% with a median time to a Response Evaluation Criteria in Solid Tumours (RECIST)-defined response of 9.6 months. Nevertheless, in the placebo arm, an ORR of 20% was observed with a median time of 13.3 months, indicating spontaneous regression. Grade 3 AEs were reported in 29% of patients receiving sorafenib, compared to 14% in the placebo group [9]. The results of this phase 3 trial supported the use of sorafenib as the primary systemic treatment option for patients with advanced or refractory DF since 2018 although not being Food And Drug Administration (FDA) or European Medicines Agency (EMA) approved. The aim of this study is to evaluate the use of sorafenib for patients with advanced and refractory DF, using real-life data from our centre.

## Methods

Between February 2020 and January 2024, 11 patients were included in this retrospective single-centre study conducted at Ghent University Hospital, which is a Belgian reference centre in sarcoma care. We reviewed medical records of patients with advanced or progressive DF who were treated with sorafenib. This study was approved by the Ethics Committee of Ghent University Hospital, and informed consent was obtained from each participant. Follow-up time was calculated from the start of treatment to the time of analysis, January 8th, 2024.

Sorafenib, obtained through samples, was initially dosed at 400 mg for patients who were symptomatic, had a critical anatomical site or had progressive disease (PD). Dose reductions and treatment delays were performed according to the recommendations in the summary of product characteristics. Dose modifications due to AEs were based on their severity with a decrease of dose level –1 (200 mg daily) and the possibility of dose reescalation. Treatment was continued until clinical or radiological progression, durable stabilisation (defined as a quiescent state without tumoral activity), unacceptable toxicity or patient refusal.

The patients underwent imaging every 12–18 weeks: for 10 patients, tumour response was assessed using magnetic resonance imaging (MRI); for one patient, with intra-abdominal DF, a computed tomography (CT) scan. Efficacy was measured according to RECIST, Version 1.1 [10]. Safety assessment of sorafenib was reported according to the National Cancer Institute’s Common Terminology Criteria for Adverse Events, version 5.0 [11].

All statistical analyses were performed using R version 4.2.2. Median treatment duration and median time to ORR were calculated using the Kaplan–Meier estimator, whereas median time to adverse event and median time to dose reduction were calculated using the Aalen–Johansen estimator with treatment discontinuation as a competing event.

## Results

**Figure 1:**
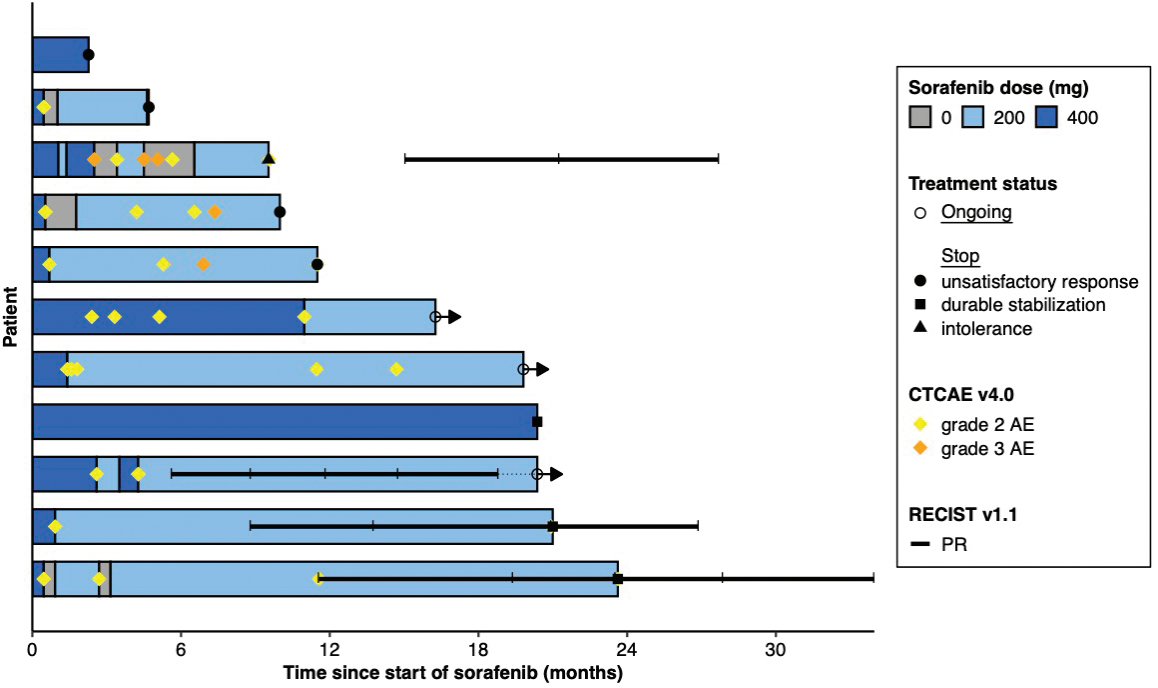
Swimmer plot: duration of response, dose and clinical outcomes of sorafenib for each patient.

Eleven DF patients with a median age of 41 years (range, 30–50 years) receiving sorafenib were enrolled in this study. 81.8% of patients reported pain. Baseline characteristics are shown in Table 1. Two thirds of the patients were female (63.6%). The rate of extra-abdominal locations was 72.7%. The median size of the tumour was 87.1 mm (range, 43.3–188.0) at baseline. Almost one third (27.3%) of patients had received previous treatment, including surgery, cryoablation and magnetic resonance-guided high-intensity focused ultrasound (MR-HIFU) ablation. Three patients initially underwent a watchful waiting approach. When active treatment was chosen, 72.7% received sorafenib as their first-line treatment. The individual patient trajectories over time are depicted in Figure 1. Median treatment duration was 20.4 months (95% CI, 10.0-NR). For three patients, treatment is ongoing. Where treatment was discontinued, the reasons were unsatisfactory response (*n* = 4), durable stabilisation (*n* = 3) and intolerance (*n* = 1).

**Table 1:** Patients baseline characteristics.

Variable	*N* = 11
**Age**	41.0 years (30.0–50.0)
**Sex**	
Female	7 (63.6%)
Male	4 (36.4%)
**Tumour site**	
Extra-abdominal	8 (72.7%)
Intra-abdominal	3 (27.3%)
**SLD**	87.1 mm (43.3–188.0)
**Pain**	9 (81.8%)
**Sorafenib line**	
1L	8 (72.7%)
2L	3 (27.3%)
**Previous therapies**	
Surgery	2 (18.2%)
MR-HIFU	1 (9.1%)
Cryoablation	1 (9.1%)

SLD: the sum of the longest diameter of the target lesions; MR-HIFU: magnetic resonance-guided high-intensity focused ultrasound.

**Figure 2:**
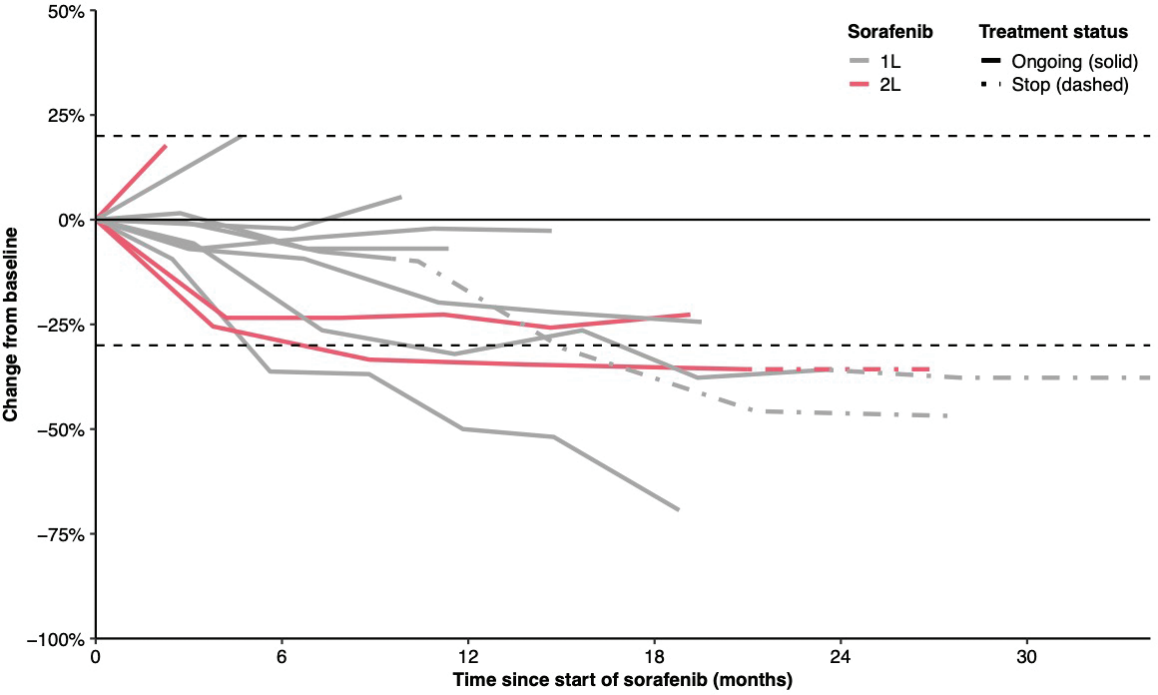
Spiderplot displaying treatment status and response of sorafenib in 11 patients with DF.

Four patients (36.3%) treated with sorafenib achieved partial response, six patients (54.5%) had a stable disease and only one patient (9.1%) had progressive disease (Figure 2). Median time to ORR was 15.0 months (95% CI, 8.8-NR). One partial response was obtained, and two partial responses were sustained after discontinuation of sorafenib (Figure 2). Four patients (36.4%) with tumours located in the groin, thorax and abdominal wall required subsequent therapy such as radiotherapy, tamoxifen or surgery.

All patients experienced grade 1 toxicity, 81.8% experienced grade 2 toxicity, 36.4% grade 3 toxicity, but no grade 4–5 toxicity was observed. The most common grade AE was skin toxicity (hand-foot syndrome, pruritus and rash) (90.9%). Nine patients (81.8%) needed a dose reduction with a median time to the first reduction of 1.1 months (95% CI, 0.5-NR), and four patients (36.4%) needed dose interruption. One patient stopped treatment due to toxicity. Median time to AE was 0.7 months for grade 1 toxicity (95% CI, 0.5–3.2), 1.4 months for grade 2 toxicity (95% CI, 0.5-NR) and NR for grade 3 toxicity (95% CI, 6.9-NR).

## Discussion

This single-centre retrospective analysis provides real-life data confirming sorafenib as an effective treatment for advanced and refractory DF. Although this disease is not malignant, the locally destructive and aggressive nature can be associated with significant morbidity, especially in a predominantly young patient population. In our study, the patients had a median age of 41 years, predominantly female and all had sporadic DF, which was mostly (72%) located extra-abdominal. This is representative of the patient population with DF [2, 4, 6].

A watchful waiting strategy is internationally accepted as a frontline approach for newly diagnosed asymptomatic patients without a critical anatomical DF location who do not progress, with close monitoring and follow-up [4–6]. In case of progressive or advanced disease, active treatment should be considered. Choosing the right treatment for the right patient at the right time remains challenging and must take into consideration tumour size and growth rate, anatomical location, risk of critical complications such as obstruction and therapy characteristics such as AEs, ORR, PFS rate and ease of administration. However, the lack of large prospective trials, the uncertainty of rare diseases and the unpredictable disease course should be acknowledged [2, 3, 6, 12], so the discussion by an experienced multidisciplinary soft tissue tumour board is needed. Active treatment can be either local or systemic, but in the absence of comparative trials, it is not possible to suggest a definitive sequence of the existing treatment options [13]. In the case of systemic therapy, randomised data only exist for pazopanib and methotrexate/vinblastine (phase 2 trial), sorafenib (phase 3 trial) and nirogacestat (phase 3 trial) [7, 9, 14]. In our study, almost one third (27.3%) of patients received prior local treatment. Nevertheless, sorafenib seemed to be the first systemic treatment option.

The use of sorafenib in patients with progressive or advanced DF has been promoted in recent years based on the results of a randomised phase 3 trial conducted by Gounder et al. [9]. They showed a PFS of 89% (95% CI, 80–99) with an ORR of 33% (95% CI, 20–48) after 1 year of treatment with sorafenib. The median time to a RECIST-defined response was 9.6 months. In our study, we achieved a similar ORR of 36.6% with a longer median time to ORR of 15.0 months (95% CI, 8.8-NR) despite the small number of patients. However, whether RECIST is an appropriate measure for efficacy in DF is debatable. Three patients with stable disease according to RECIST stopped their treatment due to durable stabilisation. Although there were no significant changes in tumour size, it was considered to be a maximum response to sorafenib. This is related to the unique histology of DF, which undergoes collagenisation as it responds to therapy or matures. These changes are reflected by a reduced T2 signal and enhancement on MRI, which are independent of change in size [1, 2, 6, 15]. There are no standard validated response criteria. The majority of prospective trials use RECIST to report efficacy, which is mainly based on the tumour size rather than biological changes [6, 15]. When assessing response, the possibility of spontaneous regression or delayed treatment effect from previous therapies must be considered. This can be difficult to assess in a clinical trial setting, but in real life, a multiparametric approach including tumour size, changes in T2 signal and clinically relevant changes is being considered to evaluate response [1, 2, 6, 15, 16]. Whether sorafenib can be rechallenged post-treatment completion is unknown, with no current data available. However, it might be a viable approach in some cases.

Although manageable, toxicity remains a concern with TKIs such as sorafenib. In our study, AEs were graded retrospectively, which has its limitations and can be biased. Most reported AEs are grade 1 and 2, although one third of patients reported grade 3 toxicity (hypertension, rash, liver toxicity). Skin toxicity is most frequently reported (87%) and is consistent with previous literature [9]. Only one patient maintained a daily dose of 400 mg. In contrast to what Gounder et al. [9] reported, where two-thirds (65%) of patients required dose interruptions and 31% of patients dose reductions, our study suggests a lower incidence of dose interruptions but a higher incidence of dose reductions, being 36.4% and 81.8%, respectively. This difference may be due to the clinical trial setting where the investigator is binded to follow the study protocol, while in real-life situations, the treating physician has more freedom in choosing between dose interruption and dose reduction in case of important AEs. Remarkably in our study, the median time to first dose reduction was only 1.1 months (95% CI, 0.5-NR). Nevertheless, the response continued even at a lower dose of 200 mg, with less toxicity, consistent with previous reports [6, 9, 17]. This raises the question of whether 200 mg daily is sufficient to achieve tumour response with less toxicity.

Our study has some limitations as it is a retrospective analysis with a small sample size, no control arm and some missing data. Most of the patients reported pain at presentation (81.8%) although clinical response was not consistently documented. Finally, sorafenib as the preferred systemic treatment option for progressive DF is uncertain due to the recent publication of the DeFI trial. This phase 3 randomised trial evaluated the efficacy and safety of nirogacestat, an oral gamma-secretase inhibitor, in patients with progressing DF. The results showed that the proportion of being event free at 1 year was 85%, with an ORR of 41% and a median time to response of 5.6 months [7]. Based on these data, nirogacestat was approved by the FDA in 2023. EMA approval is pending. A head-to-head comparison between sorafenib and nirogacestat has not yet been conducted. However, in clinical practice, the toxicity profile and patient preferences should be taken into consideration.

In conclusion, sorafenib remains an effective treatment for the challenging and evolving management of DF.

## Data Availability

The data are not publicly available due to their containing information that could compromise the privacy of research participants.
